# *In vivo* analysis of CRISPR-edited germinal center murine B cells

**DOI:** 10.3389/fimmu.2024.1473760

**Published:** 2024-10-17

**Authors:** Timothy Chege Kuria, Andrea Schneider, Favoured Baraka, Jana Wanzek, Lisa Vogg, Stefanie Brey, Katharina M. Habenicht, Thomas H. Winkler

**Affiliations:** Department of Biology, Division of Genetics, Nikolaus-Fiebiger-Center for Molecular Medicine, Friedrich-Alexander-Universität Erlangen-Nürnberg (FAU), Erlangen, Germany

**Keywords:** germinal center, CRISPR, B cells, selection, Fas, 40LB cells, high-affinity

## Abstract

The germinal center (GC) reaction is crucial for somatic hypermutation, affinity maturation, and the selection of high-affinity B cells, all of which are hallmarks of the humoral immune response. Understanding the distinct roles of various B cell genes is essential for elucidating the selection mechanisms within the GC reaction. Traditionally, studying B cell gene function in the GC reaction involved generating knock-out mice, a highly time-consuming method that necessitates complex vectors. The advent of Clustered Regularly Interspaced Short Palindromic Repeats (CRISPR) technology has simplified the creation of knock-out mice. However, even with CRISPR, the generation of knock-out mice still faces challenges, including being time-consuming, costly, having low knock-out efficiency, and raising ethical concerns regarding animal use. To address these challenges, we developed an alternative method to traditional knock-out mouse generation. Our approach entails the *ex vivo* CRISPR editing of B cells from transgenic donor mice with different B cell receptor affinities followed by their adoptive transfer into recipient mice. We present a cost-effective, rapid, versatile, and adaptable CRISPR-Cas9 method for *in vivo* loss-of-function studies of individual murine B cell genes within the context of the GC reaction. This method provides a valuable tool for investigating the complex roles of different B cell genes in the GC selection process. As proof of concept, we validated our approach by examining the role of the pro-apoptotic gene Fas in the GC selection process. We adoptively transferred a mix of Fas knock-out (Fas^KO^) low-affinity B cells, Fas wild-type (Fas^WT^) low-affinity B cells, and Fas^WT^ high-affinity B cells into recipient mice. From our results, Fas^KO^ low-affinity B cells were still outcompeted by the Fas^WT^ high-affinity B cells for selection in the GC. An important observation was the accumulation of Fas^KO^ low-affinity GC B cells when compared to the Fas^WT^ low-affinity B cells, which suggested a role of Fas in the GC selection process.

## Introduction

B cells play a pivotal role in the humoral immune response through their secretion of antibodies, which are the soluble forms of the B cell receptors (BCRs). The germinal center (GC) reaction is essential for enhancing BCR affinities, thereby enabling the production of high-affinity antibodies (1). This reaction is meticulously regulated by intricate gene networks. Understanding the specific roles of these genes within the GC context is vital for comprehending the humoral immune system (2). However, the multitude of genes involved in the GC reaction makes studying individual B cell genes challenging. One effective method to investigate gene function is through the generation of knock-out mice which lack the expression of a specific gene. Since the creation of the first B cell-specific gene knock-out mouse in 1991, this approach has been extensively utilized to characterize individual B cell genes (3). Nevertheless, the traditional method of generating knock-out mice is both costly and labor-intensive, requiring sophisticated vectors and the genetic manipulation of embryonic stem (ES) cell lines (4, 5).

The emergence of novel genetic manipulation techniques simplified the generation of knock-out mice. One such technique is Clustered Regularly Interspaced Short Palindromic Repeats (CRISPR), which utilizes endonucleases to cleave DNA at specified gene loci. The resulting double-strand breaks are imperfectly repaired via non-homologous end joining, which leads to loss-of-function mutations in the targeted genes (6, 7). This abolished the need for complex vectors as CRISPR-associated ribonucleoproteins (RNP) coupled to easily synthesized homologous sequences could now be utilized (8). Although the use of CRISPR to generate knock-out mice addressed some of the challenges associated with the conventional ES-cell approach, the generation of homozygous mutants for analyses still demands dedicated expertise (9) and usually requires at least 6 months to generate mouse models with the desirable genotype for concomitant phenotypic investigation. Also, the risk of off-target effects arising from CRISPR-mediated gene knock-outs cannot be excluded as incorrect integration of the homologous sequence could occur at unintended genomic locations (10). Furthermore, not all genes are amenable to being knocked out, especially those essential for early development. For instance, genetic ablation of genes crucial for embryogenesis may result in embryonic lethality, making it difficult to study their function (11). Another potential setback could be mosaicism, as the knock-out allele may not be present in every mouse cell (12). Chimeric mice, a common outcome of blastocyst injection, can also complicate experiments, requiring further breeding to achieve germline transmission of the gene modification, thus prolonging the entire process (13).

Ultimately, ethical concerns arise, emphasizing the need to honor the inviolability of life. Sacrificing many mice solely due to an undesired genotype is unethical (14). Thus, every conceivable effort should be made to prevent this outcome.

The use of *in vitro* models to study the GC reaction has been invaluable, reducing the need for knock-out mouse models for each gene of interest. An example is the induced-GC (iGC) culture system, which employs 40LB cells, a murine fibroblast cell line that stably expresses CD40L and BAFF, which are important B cell co-stimulatory molecules (15). Co-culturing B cells with 40LB cells drives the B cells to acquire a GC-like phenotype characterized by their expression of key GC B cell markers. Using the iGC culture system, Rajewsky and colleagues employed a small-scale CRISPR-Cas9 *in vitro* screen of primary B cells to identify genes crucial for B cell activation and differentiation into antibody-secreting cells (16). Similarly, Wöhner and colleagues applied this system to identify genes essential for the development of antibody-secreting cells (17). However, accurately replicating the physiological relevance of a gene *in vitro* remains challenging, mainly due to the incapability of *in vitro* models to imitate complex *in vivo* environments. As such, the iGC model cannot duplicate tissue microenvironment signals, including growth factors, chemokines, cytokines, and cell contact-dependent interactions which are usually present *in vivo* in a GC.

A more robust approach to studying the GC reaction is the use of *in vivo* mouse models. A few landmark studies have demonstrated the feasibility of manipulating B cells *ex vivo* and then adoptively transferring them into recipient mice. Three independent studies have shown that CRISPR-modified B cells, once transferred into recipient mice, can express broadly neutralizing antibodies (bNAbs) against HIV and actively participate in the GC reaction (18–20).

Building on these approaches, our aim was to create a CRISPR-Cas9 method to study the loss-of-function of individual murine B cell genes in the context of a GC reaction *in vivo*, specifically in a competitive system. Thus, we performed *ex vivo* CRISPR editing of B cells from transgenic donor mice with different B cell receptor affinities for a defined antigen. These edited B cells can then be adoptively transferred into recipient mice that are non-responsive to the same antigen, and upon immunization, allow for the *in vivo* generation and analysis of only donor B cell-derived GCs. To validate our approach, we sought to understand the role of the pro-apoptotic gene Fas in the GC selection process.

Fas belongs to the tumor necrosis factor receptor family, and its interaction with the Fas ligand initiates apoptosis in a wide range of cells. When this apoptotic pathway is disrupted by a lymphoproliferation mutation in the Fas gene, it results in lymphoproliferative disorders, including the development of clonally expanded, autoreactive B cells that carry somatic mutations in their receptors (21). The actual role of Fas in the GC selection process has, to some extent, been inconclusive. Using a lymphoproliferation mouse model, Smith and colleagues proposed that while Fas is highly expressed in GC B cells, it is not required to regulate B cell responses to antigens, including selecting high-affinity B cells and maintaining B cell tolerance. This suggests that other pathways, possibly independent of Fas, regulate the immune response in the GC (22). On the other hand, a classical study by Hao and colleagues suggested that the presence of Fas in GC B cells is crucial for preserving immune homeostasis. Without Fas, the selection process within GCs becomes compromised, resulting in unchecked lymphocyte proliferation and severe immune disorders (23).

The selection process in the GC is affinity-mediated, and from our data and that of others, high-affinity B cells are always positively selected at the expense of the low-affinity B cells in the GC (24). Consequently, since Fas is upregulated in GC B cells (18), we hypothesized that Fas might play a role in the elimination of low-affinity GC B cells. To investigate this, we put Fas knock-out (Fas^KO^) low-affinity B cells in competition with both Fas wild-type (Fas^WT^) low-affinity B cells and Fas^WT^ high-affinity B cells in recipient mice and studied the ensuing GC response. Both Fas^KO^ low-affinity GC B cells and Fas^WT^ low-affinity GC B cells were outcompeted by Fas^WT^ high-affinity GC B cells. Interestingly, when comparing Fas^KO^ low-affinity GC B cells and Fas^WT^ low-affinity GC B cells, we observed an increase in the proportion of Fas^KO^ low-affinity GC B cells relative to the Fas^WT^ low-affinity GC B cells suggesting a potential role of Fas in the GC selection process.

## Materials and equipment

### Equipment

Neon^®^ Transfection System starter pack: ThermoFisher Scientific, cat # MPK5000.

Flow cytometer and general lab supplies include but are not limited to pipettes and cell culture flasks.

### General materials

40LB cells: BALB/c3T3 cells stably expressing mouse CD40L and mouse BAFF (15).
Mouse models: B1-8 (B1-8^lo^ and B1-8^hi^) (25) and 33.C9gl mice (26).
3. B cell medium: RPMI-1640 supplemented with 10% FCS, 5 mM L-glutamine, and 0.02 mM 2-Mercaptoethanol.
B cell stimulation medium: RPMI-1640 supplemented with 10% FCS, 5 mM L-glutamine, 0.02 mM 2-Mercaptoethanol, and 5 µg/mL CpG.
Phosphate buffered saline (PBS): ThermoFisher Scientific, cat # J61196.AP.
Washing buffer: PBS supplemented with 0.5% FCS and 5 mM EDTA.
CpG (ODN1826): Integrated DNA Technologies, 5’-TCCATGACGTTCCTGACGTT-3’, phosphorothioate bonds.
Recombinant murine IL-4: Peprotech, cat # 214-14.
B cell isolation kit, mouse: Miltenyi Biotech, cat # 130-090-862.
CD19 MicroBeads, mouse: Miltenyi Biotech, cat # 130-121-301.
Monarch^®^ Genomic DNA Purification Kit: New England Biolabs, cat # T3010L.
4-Hydroxy-3-nitrophenyl acetyl (NP) hapten conjugated to Keyhole Limpet Hemocyanin (KLH): BioCat, cat # N-5060-25-BS.
Keyhole Limpet Haemocyanin (KLH): Merck, cat # H7017-20MG.
Alum Adjuvant: Thermo Scientific™ Imject™, cat # 77161.

### CRISPR components

1. Alt-R™ S.p. HiFi Cas9 Nuclease V3: Integrated DNA Technologies, cat #1081061.
2. tracrRNA: Integrated DNA Technologies, cat # 1072534.
3. Nuclease-free duplex buffer: Integrated DNA Technologies, cat # 11-01-03-01.
4. 1X IDTE buffer: Integrated DNA Technologies, cat #11-01-02-02.
5. Alt-R™ Cas9 Electroporation Enhancer: Integrated DNA Technologies, cat # 1075916.

### Primers and crRNAs

1. B2M exon-targeting crRNA: Integrated DNA Technologies, Design ID: Mm.Cas9.B2M.1.AG, crRNA target sequence: AGTATACTCACGCCACCCAC.
2. B2M intron-targeting crRNA: Integrated DNA Technologies (IDT), crRNA target sequence: TGGTGCATACTAAGTGTCAA.
3. Fas crRNA: Integrated DNA Technologies, crRNA target sequence: TCAGAAGGATTATATCAAGG.
4. Fas 5´ NGS sequencing primer (forward): Integrated DNA Technologies, ACACTCTTTCCCTACACGACGCTCTTCCGATCTCAACCACCAACATGCCTTGGTTT.
5. Fas 3´ NGS sequencing primer (reverse): Integrated DNA Technologies, GACTGGAGTTCAGACGTGTGCTCTTCCGATCTAGTCCTGCTCCCCCTTCTTTGTAA.

### Antibodies

1. APC/Cyanine7 anti-mouse CD19 Antibody, RRID: AB_830706 (BioLegend, cat # 115529).
2. APC anti-mouse CD45.1 Antibody, RRID: AB_313503 (BioLegend, cat # 110714).
3. FITC anti-mouse CD45.2 Antibody, RRID: AB_313443 (BioLegend, cat # 109806).
4. Biotin anti-mouse/human GL7 Antigen (T and B cell Activation Marker) Antibody, RRID: AB_2721505 (BioLegend, cat # 144616).
5. Brilliant Violet 510™ Streptavidin: Biolegend, cat # 405233.
6. PE anti-mouse CD38 Antibody, RRID: AB_3068272 (BioLegend Cat. No. 165610).
7. PE-Cy™7 hamster anti-mouse CD95, RRID AB_396768 (BD Biosciences, cat # 557653).
8. FITC MHC Class I (H-2Kb) Monoclonal Antibody (AF6-88.5.5.3), RRID: AB_11149502 (eBioscience™, cat # 11-5958-82).

### Software and tools

Geneious Prime 2022.2.2 (www.geneious.com) for gRNA design, FlowJo v10 (www.flowjo.com) for analysis of flow cytometry data. Figures were generated using a licensed version of Biorender. Graphs and statistical analysis were performed using GraphPad Prism v10.

## Methods

### Isolation and stimulation of splenic B cells

B cells from the donor mice were mechanically extracted from the spleen and resuspended in washing buffer. Following erythrocyte depletion, B cells were enriched using the B cell isolation kit. Untouched B cells were then cultured for 24 hours in B cell stimulation medium, which is critical as RNP transfection does not work on resting cells. In our setup, 5 μg/mL of CpG was used to stimulate B cells as it does not necessarily differentiate the cells but rather puts them in cell cycle (27).

### Adoptive transfer of B1-8^lo^ and B1-8^hi^ B cells

**Figure 1:**
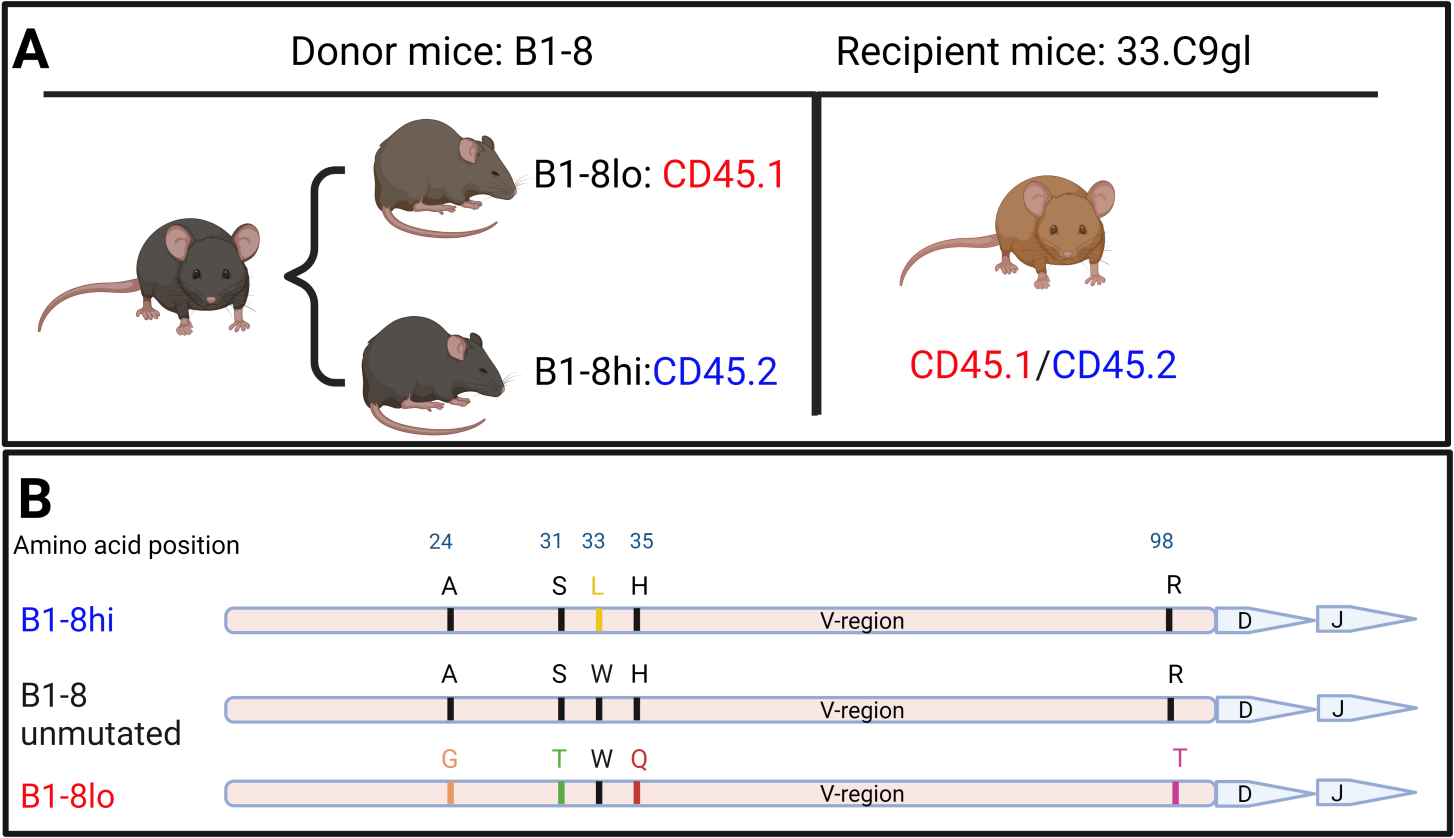
**(A)** Iterations of B1-8 mice used as sources of donor B cells as well as recipient mice used for adoptive transfers. **(B)** Graphical representation of mutations on the immunoglobulin heavy chain compared to those on the original B1-8 heavy-chain variable (V) region that led to either an increased or reduced binding affinity to the NP hapten.

For adoptive transfer experiments, we used CD45.1 B1-8^lo^ and CD45.2 B1-8^hi^ mice, both obtained from M. Nussenzweig through F. Nimmerjahn (FAU, Erlangen), as well as CD45.1/CD45.2 heterozygous 33.C9gl mice (**Figure 1A**).

As donor mice, we used B1-8 mice, which have an immunoglobulin heavy chain knock-in derived from a 4-hydroxy-3-nitrophenyl (NP) hapten binding antibody with an affinity (K_a_) of 5×10^5^ M^−1^. A tryptophan to leucine substitution at codon 33 of the B1-8 heavy chain results in a tenfold increase in NP affinity (K_a_ = 5×10^6^ M^−1^, referred to as B1-8^hi^, **Figure 1B**). Conversely, amino acid substitutions with glycine, threonine, glutamine, and threonine at position 24, 31, 35, and 98, respectively, decrease NP-binding by a factor of four (K_a_ = 1.25×10^5^ M^−1^, denoted as B1-8^lo^, **Figure 1B**) (25, 28, 29). These two iterations of B1-8 mice, which we used as our donor B cell mice, were bred to be on different allelic variants of the pan-hematopoietic cell marker CD45: B1-8^lo^ on CD45.1 and B1-8^hi^ on CD45.2.

For B cells from B1-8 mice to bind the NP hapten in a subsequent immune response, they must pair with a lambda light chain during VDJ recombination. If they pair with a kappa light chain instead, NP binding is unlikely to occur (30). With this in consideration, we used an additional in-house mouse model, 33.C9gl, as recipient mice. 33.C9gl mice have a heavy chain and kappa light chain knock-in of an anti-DNA antibody derived from a systemic lupus erythematosus patient (26). Because these recipient mice have both a transgenic human heavy chain and a kappa light chain, they cannot mount adequate B cell responses to the NP hapten, justifying them as our NP non-responsive recipient mice.

**Figure 2:**
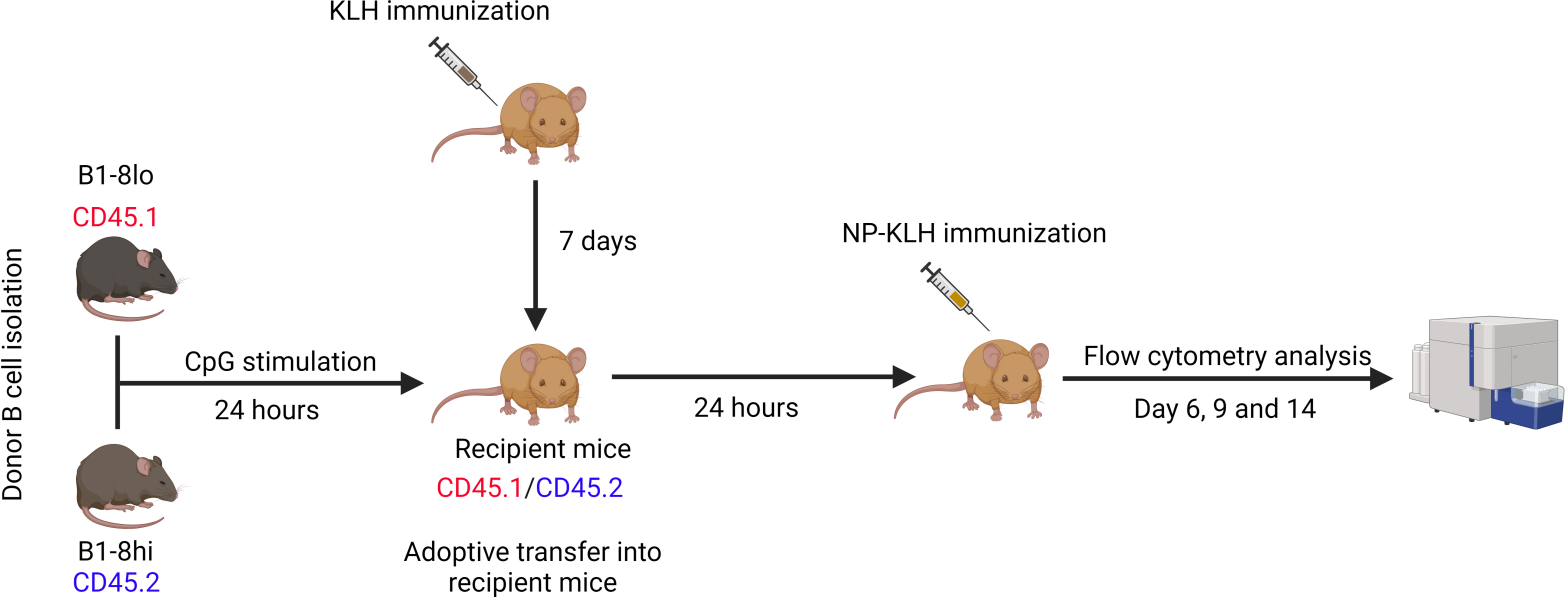
Adoptive transfer of competition experiments. B cells were isolated from B1-8^lo^ and B1-8^hi^ mice, mixed in predetermined proportions, and cultured in B cell stimulation medium for 24 hours before being adoptively transferred into KLH-primed recipient mice. 24 hours later, the recipient mice were immunized with 100 μg alum adjuvanted NP-KLH and sacrificed 6, 9, or 14 days later for flow cytometry analysis of the GC response.

Donor B cells from B1-8^lo^ and B1-8^hi^ mice were cultured for 24 hours in B cell stimulation medium, mixed in predetermined proportions, and adoptively transferred into 33.C9gl mice immunized with KLH with alum as an adjuvant a week before adoptive transfers. The KLH immunization serves as a T cell prime, which enables a much more robust T cell-mediated immune response upon NP-KLH immunization. After 24 hours, the recipient mice were immunized with 100 μg of alum-adjuvanted NP-KLH and then sacrificed on days 6, 9, or 14 to study the GC response (**Figure 2**).

### CRISPR-Cas9 approach

**Figure 3:**
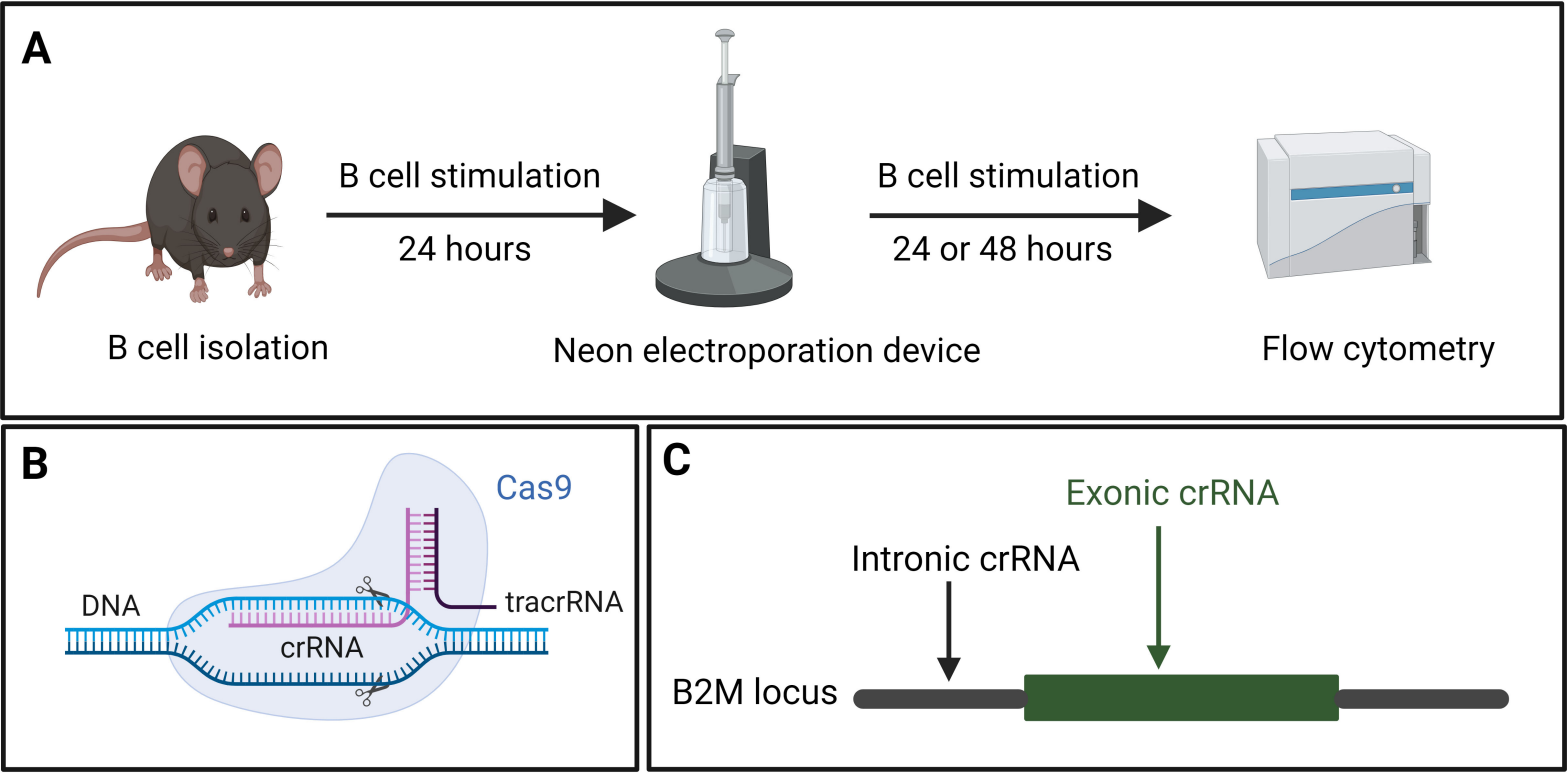
**(A)** Splenic B cells were isolated from mice and cultured in B cell stimulation medium for 24 hours, followed by CRISPR-mediated gene knock-out using the Neon electroporation device. Electroporated B cells were returned to the B cell stimulation medium for 48 hours. The efficiency of the knock-out was analyzed using flow cytometry. **(B)** Components of CRISPR showing the crRNA, target DNA, tracrRNA as well as the Cas9 protein. **(C)** Graphical representation of the exonic and intronic crRNA used to target a gene of interest, using B2M as an example.

Our approach utilized the CRISPR toolbox, primarily consisting of two essential elements: CRISPR-associated (Cas9) proteins and a guide RNA (gRNA). The gRNA comprises two synthetic subcomponents: a trans-activating CRISPR RNA (tracrRNA) that is annealed to a CRISPR/targeting RNA (crRNA). Combining the Cas9 proteins with gRNAs generates RNPs that were then used to transfect B cells. A Neon Electroporation Device™ was used to electroporate B cells with the RNPs of predesigned crRNA. Electroporation efficiency was then determined by flow cytometry or next-generation amplicon sequencing (**Figures 3A, B**).

The *in-silico* design of the crRNA followed three rules. First, preference was given to crRNAs that target the first exons of the Coding Sequence (CDS). Secondly, the crRNAs should have relatively high on-target efficiency. Finally, they should have a low predicted off-target activity. Since RNPs are quickly degraded by cells, off-targets are not a concern in an RNP approach (31). Once appropriate crRNA sequences had been identified, they were commercially sourced. To reduce the cost of experiments, tracrRNA was bought in bulk.

To generate 100 μM gRNA, 10 nmol crRNA were resuspended in 50 μL nuclease-free duplex buffer to achieve a final concentration of 200 μM. Subsequently, 50 μL of 200 μM tracrRNA were added to the crRNA. The resulting mixture was denatured at 95°C for 5 min and then slowly cooled to facilitate annealing. As control experiments, a custom-design crRNA targeting an intron was also utilized (**Figure 3C**).

### Transfection of stimulated B cells using the Neon Electroporation Device™

The transfection components were assembled as follows: Electrolysis Buffer E1 and Transfection Buffer T were allowed to reach room temperature. To create an RNP complex suitable for transfecting 2x10^6^ B cells, 186 pmol Cas9 (equivalent to 3 μL from a 62 μM Cas9 solution) were combined with 220 pmol pre-designed gRNA (equivalent to 2.2 μL from the 100 μM gRNA solution). 4.8 μL 1X IDTE Buffer was then added to this mixture and incubated at room temperature for 15 minutes to allow the formation of an RNP.

Next, stimulated B cells were washed with pre-warmed PBS. The cells were then counted and resuspended in Buffer T to achieve a final concentration of 40x10^6^ cells/mL. To 53 μL of Buffer T, 10 μL of the RNP complex, 5 μL of 100 μM Cas9 Electroporation Enhancer, and 50 μL of cells resuspended in Buffer T were added (**Table 1**). This mixture was then incubated for 5 minutes at room temperature.

**Table 1:** Electroporation components required to electroporate 2x10^6^ B cells.

Components	gRNA	Cas9	IDTE buffer	Electroporation enhancer	Buffer T	Cells	Total volume
**Concentration**	100 μM	62 μM	*NA*	100 μM	*NA*	40x10^6^ cells/mL	
**Volume**	2.2 μL	3 μL	4.8 μL	5 μL	53 μL	50 μL	**118 μL**

Using the Neon Electroporation Device, 100 μL of the B cell mix were electroporated using a single 20 ms, 1600 V pulse. The B cells were then immediately transferred to pre-warmed B cell stimulation medium and incubated at 37°C. Phenotyping or adoptive transfers were done after 24 or 48 hours.

### Analysis of CRISPR-based gene edits

Both flow cytometry and sequencing-based approaches were employed to assess the efficiency of CRISPR-based genome edits. Flow cytometry was performed to analyze surface molecules such as the MHC class I molecule or the Fas receptor. In scenarios where flow cytometry was impractical or the need for an additional confirmatory approach arose, sequencing-based approaches were also used.

Next-generation amplicon sequencing was employed to quantify gene editing efficiency in knock-out cells. This analysis utilized the Amplicon-EZ commercial service from Genewiz (Azenta Life Sciences). Following PCR amplification, the amplicons were normalized to a 20 ng/µL concentration and purified. Purified PCR products ranging from 150 to 500 base pairs containing partial Illumina^®^ adapter sequences were submitted for sequencing.

Additionally, a reference sequence and the sequences of primer binding sites were provided to facilitate mutation quantification through paired-end Illumina sequencing. Quality control, adapter sequence trimming, reference sequence alignment, and mutant sequence quantification were performed using NGS Genotyper v1.4.0 (Illumina Inc.).

### Adoptive transfer of CRISPR-edited B1-8^lo^ and wild-type B1-8^hi^ B cells

Each experiment consisted of an experimental arm and a control arm. In the control arm, wild-type (WT) B1-8^lo^ B cells mixed with WT B1-8^hi^ B cells were adoptively transferred into the recipient mice. In the experimental arm, while maintaining the same ratio of B1-8^lo^ to B1-8^hi^ B cells, the gene-of-interest was knocked out in a proportion of B1-8^lo^ B cells. In the case of Fas knock-out experiments, the starting proportion of knock-out B cells was intentionally adjusted to around 40% by adding WT B1-8^lo^ B cells to the electroporated cell mix to enable the detection of an increase in the proportions of B1-8^lo^ CRISPR-edited GC B cells.

B cell mixtures for the control and experimental arm were stimulated for 48 hours, followed by adoptive transfer into KLH-primed 33.C9gl recipient mice. These recipient mice were immunized 24 hours later with alum-adjuvanted NP-KLH, and the GC response was subsequently analyzed on days 6, 9, and 14 post-immunization.

**Figure 4:**
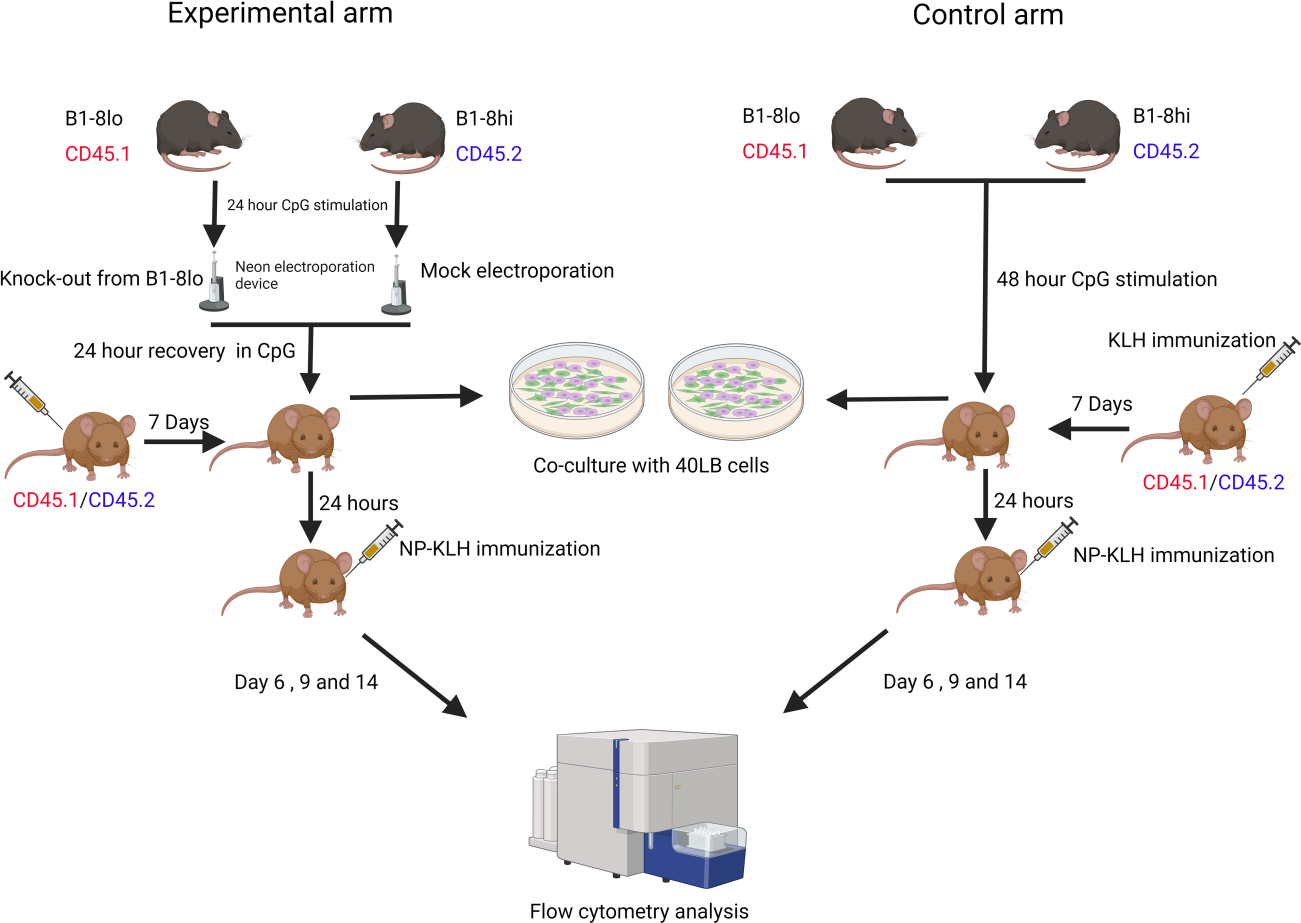
Experimental workflow for both the control and experimental arms. Control arm: B1-8^lo^ and B1-8^hi^ B cells were isolated, mixed in predetermined proportions, and then cultured in B cell medium for 48 hours before being adoptively transferred into KLH-primed recipient mice. 24 hours later, the recipient mice were then immunized with alum-adjuvanted NP-KLH and sacrificed 6, 9, or 14 days later for flow cytometry analysis of the GC response. Experimental arm: B cells from both B1-8^lo^ and B1-8^hi^ mice were isolated and stimulated for 24 hours. B1-8^lo^ B cells were electroporated with a Fas targeting crRNA while the B1-8^hi^ B cells were mock electroporated. B1-8^lo^ and B1-8^hi^ B cells were mixed in predetermined proportions and stimulated for 24 hours before being adoptively transferred into KLH-primed recipient mice. 24 hours later, the recipient mice were immunized with alum-adjuvanted NP-KLH and sacrificed 6, 9, or 14 days later for flow cytometry analysis of the GC response. On the input day, for both the control and experimental arm, flow cytometry was performed to get an exact proportion of B1-8^hi^ and B1-8^lo^ donor B cells. Other aliquots of the input B cells from the control and experimental arm were also co-cultured with 40LB cells to enable a more precise determination of the proportion of Fas expressing input B cells.

**Figure 5:**
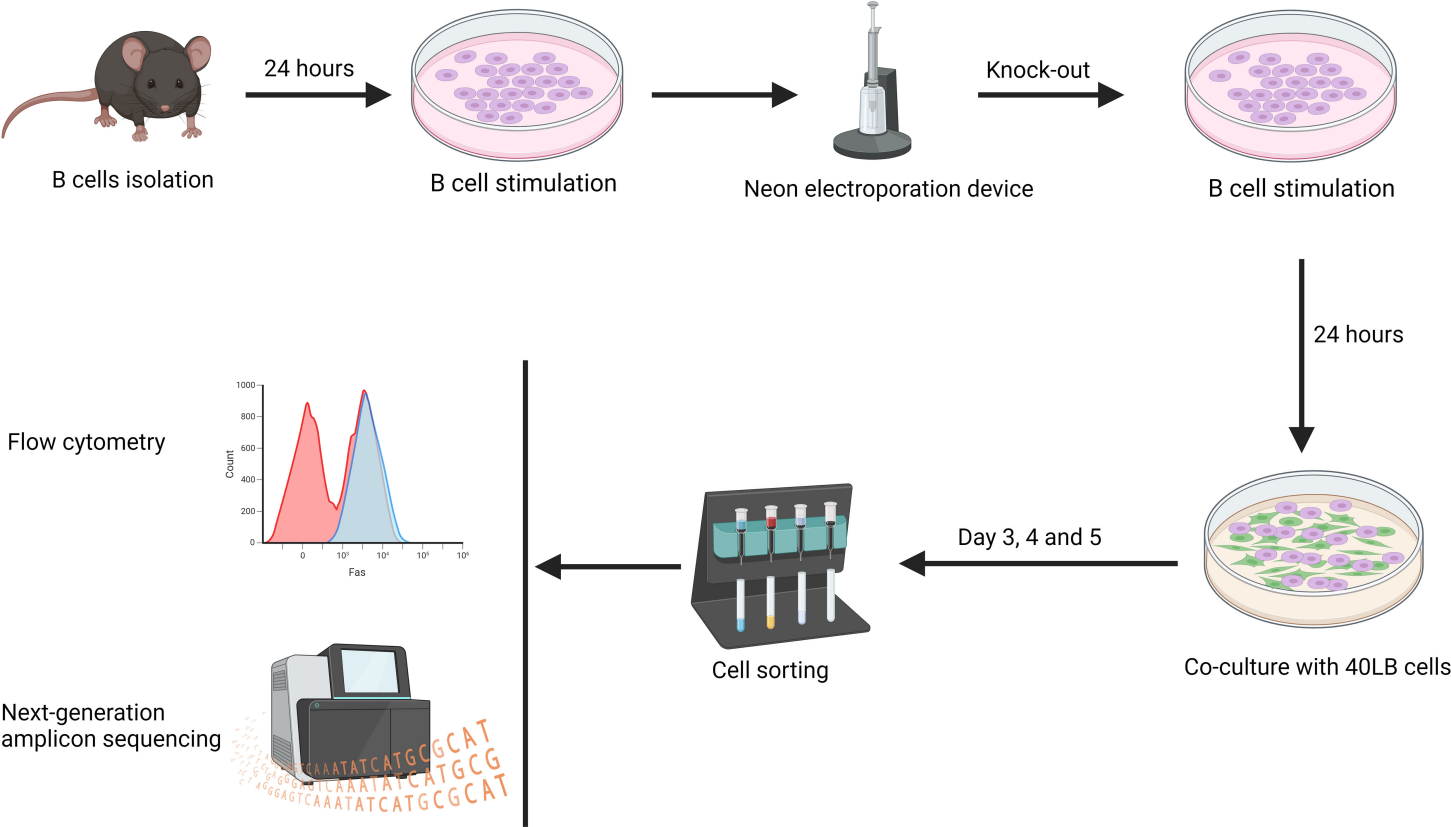
Splenic B cells were isolated and cultured in B cell stimulation medium for 24 hours. Subsequently, the Fas gene was knocked out in a proportion of the B cells, which were cultured for 24 hours. The B cells were then co-cultured with 40LB cells for 3, 4, and 5 days. On these days, flow cytometry analysis was performed directly on the co-culture. For next-generation amplicon sequencing, 40LB cells were first depleted using CD19 microbeads before DNA was extracted from iGC B cells (n=2 - 3/time-point).

To assess the efficiency of knocking out Fas in the input B cells, the 40LB culture system was utilized to achieve a GC-like state since resting B cells and CpG-activated B cells do not express high levels of Fas. Aliquots of the input B cells from the experimental and control arm were co-cultured with 40LB cells for 5 days to enable a phenotypic quantification of Fas knock-out efficiency (**Figure 4**). 1x10^5^ of the input B cells were co-cultured for 5 days with radiation-inactivated 40LB cells in B cell medium supplemented with 1 ng/mL IL-4. An *in vitro* time-series experiment was also performed to confirm that Fas^KO^ B cells had no competitive advantage or disadvantage over Fas^WT^ B cells (**Figure 5**).

### *In vitro* study of CRISPR-edited B cells

To confirm that Fas^KO^ and Fas^WT^ B cells had similar growth rates and that there was no *in vitro* selection of the Fas^KO^ B cells, a time-series experiment was performed. B cells were isolated from donor mice and cultured in B cell stimulation medium for 24 hours before CRISPR editing. The mix of Fas^KO^ and Fas^WT^ B cells were cultured for 24 hours in B cell stimulation medium before being co-cultured with 40LB cells. To determine the temporal effect of the gene knock-out *in vitro*, next-generation amplicon sequencing and flow cytometry was performed on days 3, 4, and 5. For next-generation amplicon sequencing, induced GC (iGCs) B cells were enriched using CD19 microbeads, and their DNA was subsequently extracted for sequencing (**Figure 5**).

## Results

### Assessment of electroporation efficiency: *in vitro* knock-out of the B2M gene

**Figure 6:**
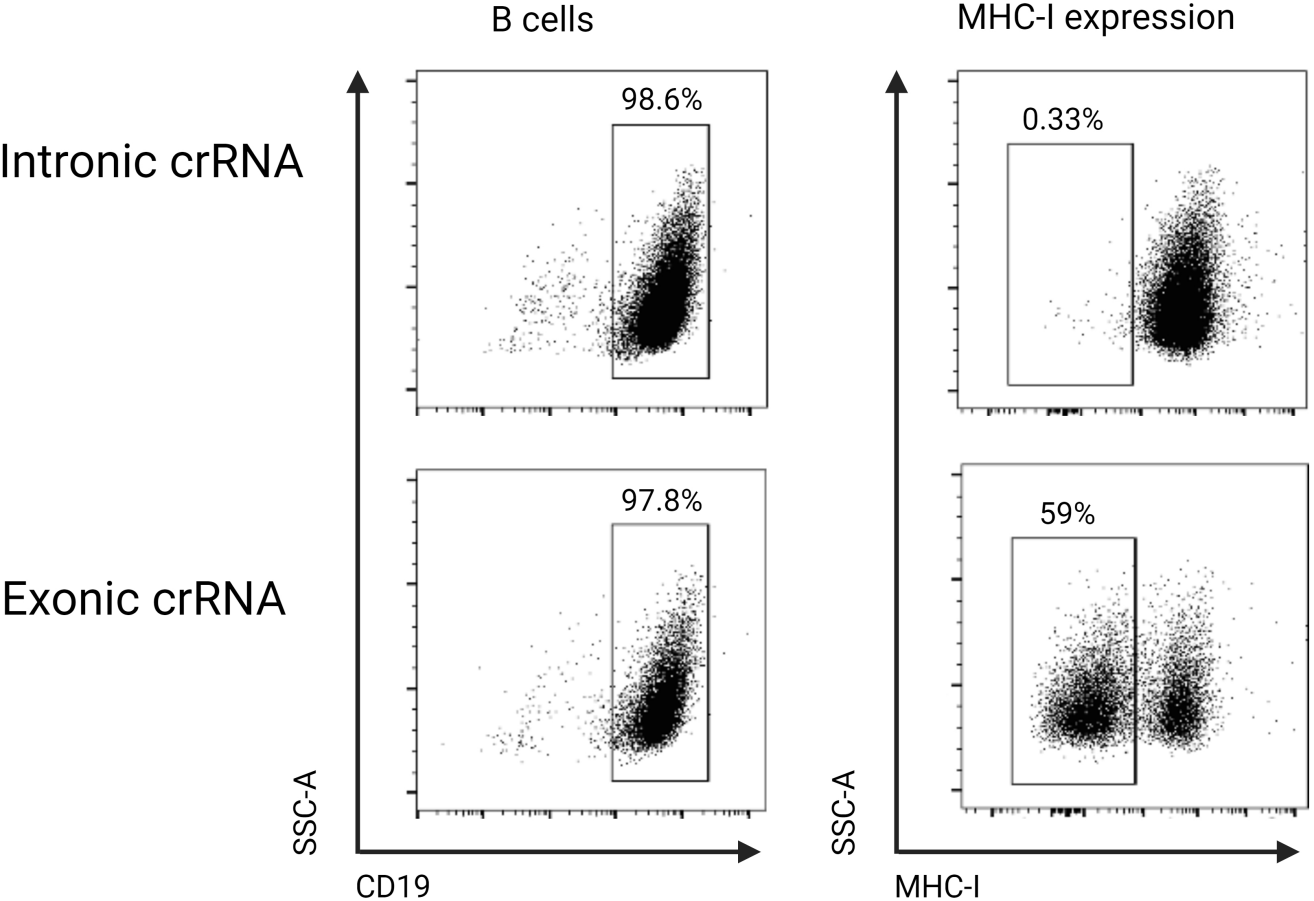
B cells were cultured in stimulation medium for 24 hours and were either electroporated with an intronic (control) or exonic B2M-targeting crRNA and phenotyped 48 hours later using flow cytometry. When the exonic crRNA was used, 59% of B cells did not have detectable cell surface expression of MHC class I, while in the control intronic targeting crRNA, there was no change in the cell surface expression of MHC class I.

To assess and potentially optimize the efficiency of our electroporation, we first designed a crRNA targeting the disruption of the MHC class I molecule. MHC class I has been shown to have a relatively short half-life, which explains why the disruption very quickly reveals a phenotypic effect (32). Due to the high polymorphism observed in the MHC class I gene in humans and mice, we targeted the conserved Beta-2-Microglobulin (B2M) gene (33). The B2M gene encodes the B2M protein, which is necessary for cell surface expression of MHC class I and the stability of the peptide-binding groove (34). To target the B2M gene locus, we used a commercially available, pre-designed crRNA targeting the exon and a custom intron-targeting crRNA as a control. We then used flow cytometry to quantify the proportion of B cells that had no detectable amount of MHC class I molecules 48 hours post-electroporation with appropriate crRNAs. We used this experiment to optimize for electroporation conditions. A single 20 ms, 1600 V pulse was found to have the optimal cell viability with the highest knock-out efficiency. In the control electroporation, all B cells had detectable expression of MHC class I, while in the B2M targeted electroporation, 59% of the B cells did not have any detectable levels of MHC class I (**Figure 6**).

### Adoptive transfer of B1-8^lo^ and B1-8^hi^ B cells into recipient mice

To study GC dynamics, we developed an experimental approach in which we could adoptively transfer high-affinity and low-affinity B cells and put them in competition with each other in recipient mice. Two iterations of the classical, NP-reactive B1-8 mouse model were employed as donor mice: a high-affinity mouse model (B1-8^hi^) and a low-affinity mouse model (B1-8^lo^) (25). For recipient mice, we adapted an in-house 33.C9gl mouse model; any mouse model carrying a non-NP-reactive B cell receptor can also be utilized.

Briefly, 24-hour stimulated 3x10^6^ B1-8^lo^ B cells and 3x10^3^ B1-8^hi^ B cells were adoptively transferred into each alum-adjuvanted KLH immunized recipient mouse. 24 hours later, the recipient mice were immunized with 100 µg alum-adjuvanted NP-KLH and sacrificed 6 or 9 days later for an analysis of the GC response.

**Figure 7:**
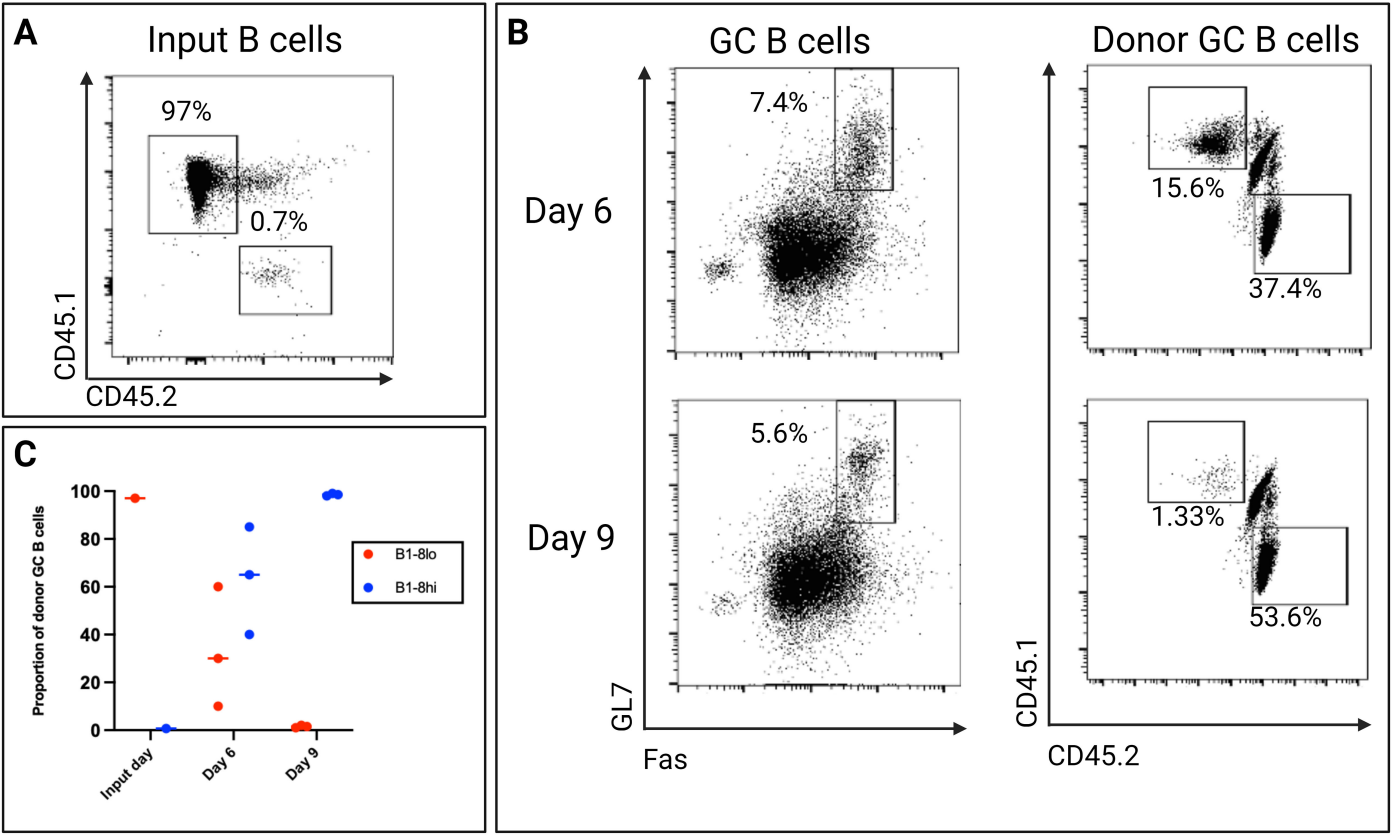
**(A)** The proportion of B1-8^lo^ (CD45.1) and B1-8^hi^ (CD45.2) B cells transferred into each recipient mouse. 3x10^6^ B1-8^lo^ B cells and 3x10^3^ B1-8^hi^ B cells were adoptively transferred to each recipient mouse, with approximately 97% coming from the B1-8^lo^ mice and only 0.7% from the B1-8^hi^ mice. **(B)** Representative day 6 and day 9 flow cytometry dot plots showing the proportion of donor GC B cells. **(C)** Scatter plot showing the proportion of donor B cells on the input day and days 6 and 9 (n=3/time-point, experiment repeated at least 3 times using different starting proportions).

Despite being significantly outnumbered at a ratio of approximately 1:100 on the input day, B1-8^hi^ B cells could still outcompete the B1-8^lo^ B cells. By day 6, the number of B1-8^hi^ B cells had already begun to surpass the B1-8^lo^ B cells in the GC reaction. This competitive advantage continued to intensify, and by day 9, B1-8^hi^ B cells had almost completely overtaken the B1-8^lo^ in the GC (**Figure 7**). It is important to note that the B1-8^lo^ B cells were always outcompeted by the B1-8^hi^ B cells for selection in the GC in multiple experiments that we conducted. We next sought to investigate what factors might mediate the observed selection *in vivo.*

### Role of Fas in the selection of high-affinity B cells in the germinal center

**Figure 8:**
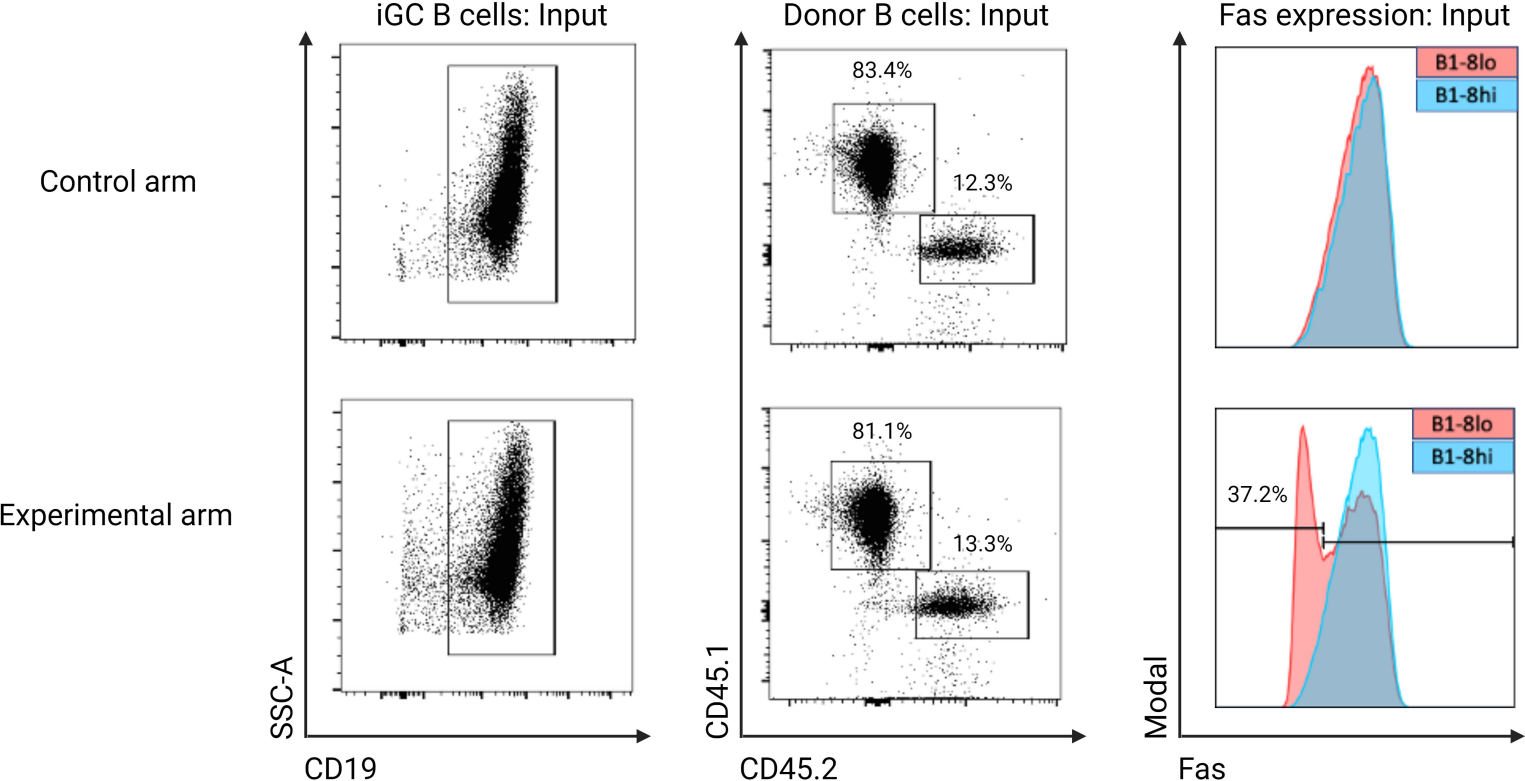
Proportion of Fas expressing input donor B cells from both the experimental and control arm that were used for adoptive transfer. Input donor B cells were co-cultured with 40LB cells to stimulate Fas expression. In the control arm, both B1-8^lo^ (CD45.1) and B1-8^hi^ (CD45.2) B cells have comparable Fas expression levels, while in the experimental arm, 37.2% of B1-8^lo^ B cells have no detectable Fas expression: This is the starting proportion of Fas knock-out B cells in the experimental arm.

Having successfully established an *in vitro* system of knocking out genes in murine B cells and an *in vivo* competition system of B cells of different affinities, we next sought to set up an adaptable *in vivo* CRISPR-Cas9 protocol that can be used to study the role of genes potentially involved in positive or negative selection in the context of a GC reaction. Since Fas is upregulated by B cells that participate in the GC reaction and its exact role in selection processes in GC cells is still not entirely clear (35), we used our experimental model to test the hypothesis that Fas plays a role in the negative selection of low-affinity B cells within the GC. Our experiment had two arms: a control arm and an experimental arm. In the experimental arm, Fas was knocked out from approximately 37.2% of the input B1-8^lo^ B cells, which were then mixed with B1-8^hi^ B cells before being adoptively transferred into alum-adjuvanted recipient mice. In the control arm, Fas^WT^ B1-8^lo^ and Fas^WT^ B1-8^hi^ B cells were mixed and then adoptively transferred into alum-adjuvanted recipient mice. In both arms, a ratio of B1-8^lo^ to B1-8^hi^ B cells of 1:6.5 was used, with a total of approximately 3x10^6^ B cells being adoptively transferred to each recipient mouse. To enable a precise determination of the proportion of Fas expressing input B cells by flow cytometry, 1x10^5^ of the input B cells were co-cultured with 40LB cells for 5 days (**Figure 8**). This 40LB culture system leads to a significant upregulation of Fas, which enabled a precise determination of the Fas knock-out efficiency, especially in the experimental arm.

As determined by the 40LB culture system, 37.2% of the input B1-8^lo^ B cells in the experimental arm were Fas knock-out cells. On the other hand, B1-8^lo^ and B1-8^hi^ input donor B cells had similar Fas expression levels in the control arm (**Figure 8**).

**Figure 9:**
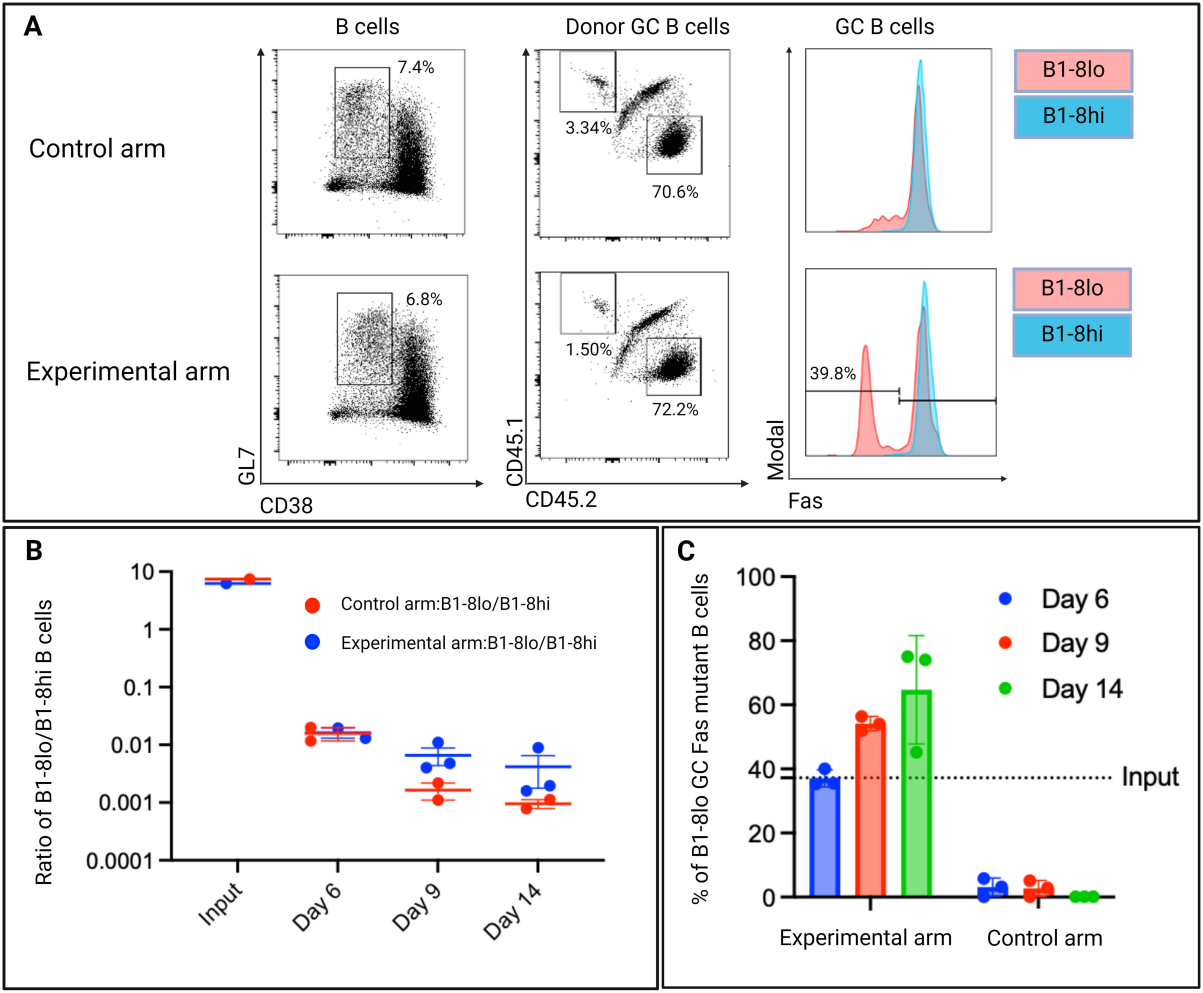
**(A)** Representative day 6 flow cytometry plots showing Fas expression levels of donor GC B cells in both the control and experimental arm. GC B cells were defined as GL7 positive and CD38 negative B cells. B1-8^hi^: CD45.2, B1-8^lo^: CD45.1. **(B)** Ratios of B1-8^lo^/B1-8^hi^ GC B cells at input day and days 6,9, and 14. **(C)** Proportion of Fas knock-out B cells in B1-8^lo^ GC B cells in the experimental arm. In the control arm, all B1-8^lo^ GC B cells expressed Fas. The dotted horizontal line depicts the input proportion of Fas^KO^ B cells as determined by 40LB culture system propagated input donor B cells (n=3/time-point, experiment repeated at least once).

Upon adoptive transfer into recipient mice, Fas^KO^ B cells were still able to fully participate in the GC reaction, showing that our stimulation and electroporation protocol did not impair the physiological activity of the gene-edited B cells (**Figure 9A**).

Examining the total donor B cells in both the control and experimental arm, Fas^KO^ B1-8^lo^ B cells and Fas^WT^ B1-8^lo^ B cells were outcompeted by the B1-8^hi^ B cells, as the B1-8^lo^/B1-8^hi^ ratio fell below 1 (**Figure 9B**).

Although the Fas^WT^ B1-8^hi^ B cells still outcompeted both Fas^KO^ B1-8^lo^ B cells and Fas^WT^ B1-8^lo^ B cells, there was a higher ratio of B1-8^lo^/B1-8^hi^ in the experimental arm when compared to the control arm at days 9 and 14 (**Figure 9B**). Focusing on the B1-8^lo^ GC B cell population in the experimental arm, there was an increase in the proportions of Fas^KO^ B1-8^lo^ GC B cells from an input proportion of 37.2% to an average of 38% on day 6 to around 55% on day 9, which reached 67% by day 14 (**Figure 9C**). This indicated that a level of Fas-mediated selection was occurring within the GC.

### *In vitro* effect of Fas knock-out on *in vitro* germinal center B cells

**Figure 10:**
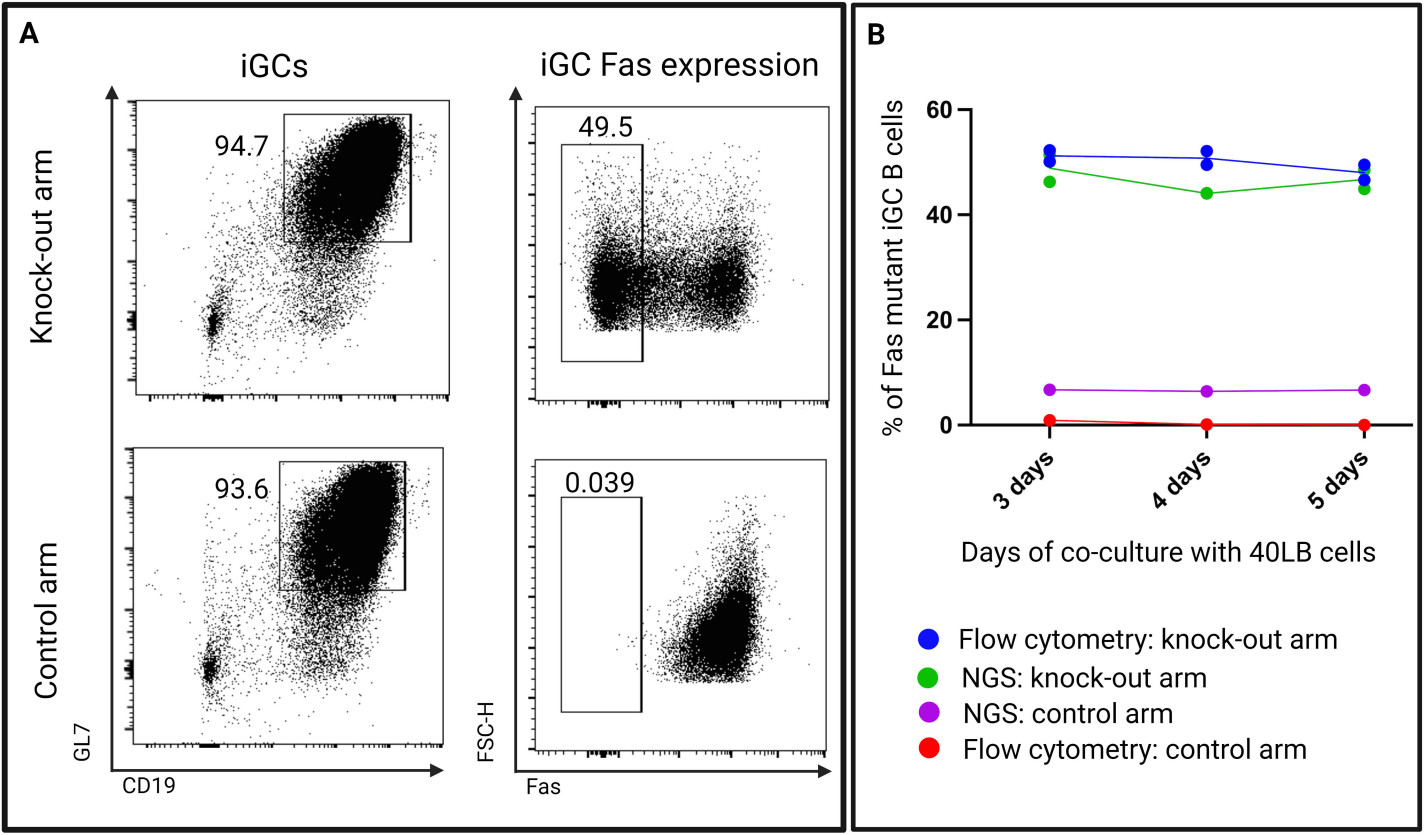
**(A)** Representative flow cytometry plots showing expression of Fas on 40LB co-cultured B cells (iGCs) from both the knock-out arm (experimental arm) and the control arm. **(B)** A comparison of Fas mutant B cell proportions determined by flow cytometry and next-generation amplicon sequencing (NGS) at days 3, 4, and 5 (n=2 - 3/time-point).

To exclude an intrinsic effect of the gene editing on cell growth, we co-cultured Fas^KO^ B cells *in vitro* with 40LB cells to induce a GC-like phenotype in the B cells. Fas^WT^ B cells were co-cultured as a control with the 40LB cells (**Figure 10A**). We performed next-generation amplicon sequencing on the extracted DNA from the iGC B cells on days 3, 4, and 5 to check for mutations in the Fas gene locus that the crRNA had targeted. The frequency of mutations was then compared to the flow cytometry knock-out efficiency.

When comparing phenotypic data from flow cytometry and next-generation amplicon sequencing data, we found a strong concordance in the frequencies of knock-out iGC B cells (**Figure 10B**). Both methods confirmed that the proportion of Fas^KO^ B cells stayed stable over the observation period. In the control arm, the 5% of the background mutation, as determined by next-generation amplicon sequencing, was possibly due to sequencing or PCR errors.

This experiment demonstrates that within 40LB cultures, the loss of Fas expression does not confer a growth advantage or disadvantage to B cells, thus excluding the likelihood of an experimental artifact in our *in vivo* data.

## Discussion

There are various delivery approaches to introduce CRISPR components into cells for targeted gene knock-out, including the use of integrating vectors such as lentiviruses, adeno-associated vectors, and plasmids, as well as non-integrating vector approaches like the use of RNPs (36). Since RNPs are non-integrating, they are rapidly degraded, leaving minimal time for any off-target activity and minimizing the induction of any immune response in recipient mice. This is unlike integrating vectors, which may trigger immune responses due to the presence of either reporter genes in the vector backbone or the Cas9 protein, which could potentially bias the interpretation of results (37). Nevertheless, a limitation of RNPs is their inefficacy in naïve B cells, thus necessitating prior activation of B cells. The choice of activation pathway is critical; in our study, we utilized CpG, which effectively induces cell cycling in B cells without significant differentiation (27), thereby facilitating the application of the RNP approach. Additionally, careful design of crRNAs is essential to ensure high on-target efficiency, focusing on targeting the first exons of the coding sequence to maximize the probability of a successful gene knock-out.

As clearly depicted by our adoptive transfer competition experiment results, the outcome of selection in the GC always remains the same; high-affinity B cells out-compete the low-affinity B cells, most efficiently between day 6 and day 9 of the GC reaction. This coincides with the presumed selection of the first wave of GC cells in the light zone (1, 24, 38). There are two proposed models that try to explain the selection processes in the light zone: the birth-limited and the death-limited selection model (24). In both proposed selection models, apoptosis appears to play an essential role in the GC selection process. In the death-limited selection model, apoptosis is thought to actively eliminate low-affinity GC B cells, and in the birth-limited selection model, a stochastic apoptosis step eliminates GC B cells regardless of their affinities. With this, we hypothesized that since Fas is a pro-apoptotic gene whose expression is upregulated in GC B cells, it might be an important mediator of selection in the GC. We therefore sought to knock-out Fas in the low-affinity B cells and put those knock-out B cells in competition with other Fas^WT^ low-affinity B cells as well as with Fas^WT^ high-affinity B cells. The hypothesis was that since it is the low-affinity B cells that are being outcompeted by the high-affinity B cells, knocking out Fas from the low-affinity B cells would essentially confer them with a competitive advantage over not only Fas^WT^ low-affinity B cells but also the Fas^WT^ high-affinity B cells.

However, our Fas knock-out experiments revealed that Fas^KO^ low-affinity B cells and Fas^WT^ low-affinity B cells were still outcompeted by Fas^WT^ high-affinity B cells to almost the same extent. Three potential explanations may account for this observation. Firstly, there may be a pre-GC affinity-dependent cell fate decision process where low-affinity B cells are preferentially directed into the extra-follicular immune response, potentially differentiating into memory B cells, while most high-affinity B cells enter the GC response, directly differentiating into antibody-secreting cells (38). This indicates that even in the absence of Fas expression, low-affinity B cells would still be competitively disadvantaged to access the GC response. This might be one explanation for why we observed a reduction of approximately 50% in the proportion of B1-8^lo^ GC B cells when compared to the input day proportion, as early as day 6 when selection is unlikely to have already taken place in the light zone. Secondly, in line with the birth-limited model, high-affinity B cells receive more selection signals within the GC than low-affinity B cells, leading to faster proliferation of high-affinity GC B cells (24). Consequently, even without Fas, knock-out low-affinity B cells would remain at a competitive disadvantage compared to high-affinity GC B cells. Lastly, other pathways beyond receptor-mediated apoptosis might be facilitating selection in the GC. Using the same experimental approach, ongoing work in our lab aims to identify novel signaling pathways that may mediate selection in the GC.

A significant observation was the higher ratio of B1-8^lo^ to B1-8^hi^ B cells in the experimental group compared to the control group. Since the only difference between the groups was the presence of Fas^KO^ B1-8^lo^ input B cells in the experimental group, this suggested that Fas may play a role in the observed difference. This is further supported by the accumulation of Fas^KO^ GC B cells in the experimental group, which increased from approximately 38% on day 6 to around 55% on day 9 and reached 67% by day 14. To confirm that the Fas-mediated selection observed between Fas^KO^ B1-8^lo^ and Fas^WT^ B1-8^lo^ cells was not an experimental artifact, we co-cultured B cells with 40LB cells, putting Fas^KO^ B cells in competition with Fas^WT^ B cells for 3, 4, and 5 days. Our results showed that Fas knock-out did not affect the cell growth *in vitro*, as the proportion of Fas knock-out B cells remained unchanged throughout the experiment, as indicated by flow cytometry and next-generation amplicon sequencing data.

Our findings align with research proposing the existence of ‘rogue B cells’. It has been shown that Fas inactivation leads to the accumulation of a population of GC B cells that have lost antigen reactivity during the GC reaction. These ‘rogue B cells’ escape negative selection and survive, undergo somatic hypermutation and differentiate into a large population of antibody-secreting cells, including autoreactive clones (35). It is conceivable that loss of the pro-apoptotic Fas gene leads to the continued survival of GC B cells with undesirable BCR mutations. Because of this, Fas might be mediating selection in the GC by helping to kill GC B cell clones that have a reduced affinity to the activating antigen or even to kill autoreactive clones. Additionally, in line with our findings, Hao and colleagues, using a B cell-specific knock-out mouse, demonstrated that Fas expression in GC B cells is essential for maintaining immune homeostasis. Its absence disrupts the selection process in GCs, leading to uncontrolled lymphocyte proliferation and fatal immune pathology (23). Using a lymphoproliferative mouse model, Takahashi and colleagues also demonstrated that by being able to differentiate memory B cells from GC B cells, mutation in the Fas gene affected clonal selection in the GC, and they suggested that Fas-mediated apoptosis may control clonal selection and the recruitment into the memory compartment (39).

Using our experimental approach, our data therefore supports a selection model in which Fas plays a role in the GC selection process by probably aiding in eliminating low-affinity B cell clones that potentially do not acquire affinity-enhancing mutations.

## Conclusion

In summary, we have developed an experimental approach to knock-out candidate genes from B cells and assess the effects of the gene knock-out *in vivo*. Our method is relatively low-cost, fast, robust, highly versatile and flexible, as only the crRNA needs to be adapted for different target genes. Our approach effectively addresses many challenges associated with generating knock-out mice for studying B cell genes within the context of GC responses. Once the transfer model is established, the turnaround time required from designing the crRNA to the actual phenotyping/genotyping of the knock-out *ex vivo* B cells typically takes 3 to 5 weeks.

Our approach facilitates the targeting of vital genes that are indispensable during embryonic development, potentially leading to either embryonic death or long-term health issues when knock-out mice are generated. With our approach, these genes can now be effectively targeted within mature B cells, allowing for a better understanding of their functions in a more natural and physiological context. Another notable advantage of our approach is the ethical aspect. Unlike traditional knock-out mouse generation, where many mice may be inadvertently sacrificed due to incorrect genotypes, our approach ensures that only two mouse models are needed to study infinite genetic perturbations within the context of the GC response.

## Limitation of the study

One limitation of our approach is that it cannot be used to study gene function in B cell development. Since we use mature splenic B cells from donor mice, applying this experimental method to investigate genes involved in the B cell development pathway would present significant technical challenges.

## Data Availability

The original contributions presented in the study are publicly available. This data can be found here: https://www.ncbi.nlm.nih.gov/sra/PRJNA1170271.
